# Application of Smart Materials in the Actuation System of a Gas Injector

**DOI:** 10.3390/ma14226984

**Published:** 2021-11-18

**Authors:** Grzegorz Mieczkowski, Dariusz Szpica, Andrzej Borawski, Saulius Diliunas, Tilmute Pilkaite, Vitalis Leisis

**Affiliations:** 1 Faculty of Mechanical Engineering, Bialystok University of Technology, 45C Wiejska Str., 15-351 Bialystok, Poland; d.szpica@pb.edu.pl (D.S.); a.borawski@pb.edu.pl (A.B.); 2 Faculty of Mechanical Engineering and Design, Kaunas University of Technology, 56 Studentų Str., LT-50240 Kaunas, Lithuania; saulius.diliunas@ktu.lt (S.D.); tilmute.pilkaite@ktu.lt (T.P.); vitalis.leisis@ktu.lt (V.L.)

**Keywords:** smart materials, piezo actuator, alternative fuel supply, gas injector

## Abstract

This paper presents the results of research related to the selection of materials for passive and active components of a three-layer piezoelectric cantilever converter. The transducer is intended for use in a low-pressure gas-phase injector executive system. To ensure the functionality of the injector, its flow characteristics and the effective range of valve opening had to be determined. Therefore, a spatial model of the complete injector was developed, and the necessary flow analyses were performed using computational fluid dynamics (CFD) in Ansys Fluent environment. The opening and closing of the injector valve are controlled by a piezoelectric transducer. Thus, its static electromechanical characteristics were found in analytical form. On this basis, the energy demand of the converter, required to obtain the desired valve opening, was determined. Assuming a constant transducer geometry, 40 variants of material combinations were considered. In the performed analyses, it was assumed that the passive elements of the actuator are made of typical materials used in micro-electromechanical systems (MEMSs) (copper, nickel, silicon alloys and aluminum alloys). As for the active components of the converter, it was assumed that they could be made of polymeric or ceramic piezoelectric materials. On the basis of the performed tests, it was found that the energy demand is most influenced by the relative stiffness of the transducer materials (Young’s modulus ratio) and the piezoelectric constant of the active component (*d*_31_). Moreover, it was found that among the tested material combinations, the transducer made of silicon oxide and PTZ5H (soft piezoelectric ceramics) had the lowest energy consumption.

## 1. Introduction

Recently, due to the functional features, strong development and application of intelligent smart materials (SMs) have been noticeable [1,2]. SMs are defined as materials whose behavior changes in a systematic way [3]. The stimulus causing the change can be a magnetic/electric field, stress or temperature. SMs include piezoelectric materials which, in combination with other materials, are commonly used as converters enabling the measurement and/or regulation of various physical quantities such as force, deformation, temperature and pressure [4,5]. The principle of their operation is based on the conversion of electrical energy into mechanical energy or vice versa [6,7]. Piezoelectric transducers are used, for example, in medicine [8,9], agriculture [10] and the automotive industry [11,12]. In the automotive industry, great emphasis is placed on reducing CO_2_ emissions. Reducing CO_2_ emissions falls under the general scope of greenhouse gases (GHGs) [13]. Various measures are used in transport vehicles to meet this expectation, such as vehicle weight reduction combined with engine displacement reduction and turbocharging [14] or the use of low-carbon fuels [15]. Despite the steadily increasing number of hybrid [16], electric [17] or H_2_-powered [18] vehicles, internal combustion engines still represent a large part of the car market, especially in the group of vehicles already in operation. The use of low-carbon fuels is currently dominated by liquefied petroleum gas (LPG) [15] and compressed natural gas (CNG) [19]. In the case of LPG, sequential vapor phase injection systems are the largest group, mainly due to their versatility [20]. Despite some problems in the conversion of spark ignition engine to LPG-fueled [21] and some discrepancies in the values of external indicators [22], the difference in fuel price determines its use. The basic rules of lower-carbon alternative fuels usage are described in many regulations, including Corporate Average Fuel Economy (CAFÉ), Alternative Motor Fuels Act (AMFA) or California Air Resources Board (CARB2020) [23,24]. Piezoelectric-driven liquid fuel injectors are widely used in the automotive industry [25,26]. Numerous experimental studies have been carried out on piezoelectric injectors mounted in diesel engines [4,27,28,29,30,31] as well as gasoline engines [32,33,34,35]. Very often, tests are performed in constant volume chambers using laser fluorescence [36]. Modern software using CFD enables researchers to carry out a number of simulations of diesel [37] or petrol [38] injector operation and to evaluate the influence of several functional parameters on external indicators or exhaust emission. Piezoelectric transducers are also used in controlling the firing pin lift of an electromagnetic injector or the qualitative assessment of small exhaust orifices [39].

Currently, newer low-pressure gas-phase injector solutions are being sought [40]. The main criteria to be met by a contemporary gas injector are short opening and closing times. They can be achieved by replacing the classical system controlling the gas injector operation (electromagnetic actuator) with a piezoelectric actuator, which, according to [41,42], reduces the reaction time by about 3 times. In transient states, some algorithms of power system control divide the fuel dose into parts, which reduce the time of one control impulse [43]. The currently used gas injectors with electromagnetic drive cannot meet the requirement of dosing at pulse times below 1 ms [40]. The authors’ concept of a gas injector with a piezoelectric actuation system [44] is presented in the first part of Section 2 of the presented work. This injector is envisaged for use in sequential low-pressure LPG vapor supply systems of internal combustion engines. For the proposed design of the injector, its simplified spatial model was created, which was subjected to flow analysis. In this way, the flow characteristics of the injector were determined, as was the effective range of valve opening. A detailed description of the method used to obtain the flow characteristics of the injector, using computational fluid dynamics (CFD) (Ansys Fluent), is provided in Section 2.2. To ensure proper control of valve opening and closing, it was necessary to develop the electromechanical characteristics of the piezoelectric actuator used (three-layer cantilever converter). The desired characteristics were found in an analytical form. The exact solution, along with the methodology by which it was obtained, is presented in Section 2.3. The elaborated electromechanical characteristics were used to determine the energy consumption of the transducer when various materials are used for its individual components. Flow and energy analyses (performed for 40 variants of material combinations) are included in the final section.

## 2. Materials and Methods

### 2.1. Research Object

**Figure 1:**
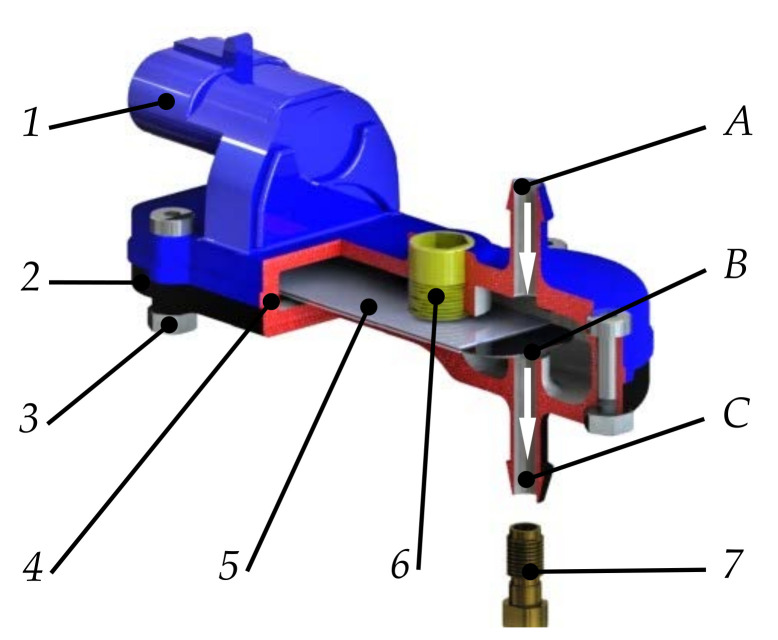
The low-pressure gas-phase injector analyzed: *1*—upper casing with electrical connection; *2*—bottom housing; *3*—screw connector; *4*—gasket; *5*—piezoelectric converter; *6*—limiter with gasket; *7*—outlet nozzle; *A*—inlet; *B*—valve; *C*—outlet.

The spatial model of the injector was developed on the basis of [44]. It should be classified as a low-pressure gas-phase injector, with its characteristic feature being a piezoelectric converter actuator. The injector (Figure 1) consists of an upper casing (1) and a lower housing (2) connected together by a screw connector (3) and a gasket (4). The piezoelectric converter (5) is fixed on one side between the housings (1) and (2).

The appearance of a control pulse in the electrical connection upper casing (1) energizes the piezoelectric converter. The deformation of the piezoelectric converter due to its one-sided mounting (cantilever) results in the displacement of the free end. As a result, valve (B) opens, allowing gas to flow from the inlet (A) to the outlet (C). An important feature of each fuel dosing subassembly is its flow characteristics and effective valve opening. The latter is regulated by a limiter (6). The limiter can have a closing spring mounted inside, which will ensure the desired closure in the non-electrically powered condition. Closing can also be achieved by inlet–outlet pressure differential or converter preload. The design of the injector provides for the possibility of limiting the flow characteristics not only by using a limiter (6) but also by fitting an outlet nozzle (7). The nozzle is commonly used in this type of component, and its inner diameter is in the range of 1.5–3 mm. The innovation in the case of the described injector, apart from the use of the piezoelectric converter itself, is the possibility of controlling not only the opening but also the closing of the valve.

### 2.2. Flow Characteristics of the Injector

**Figure 2:**
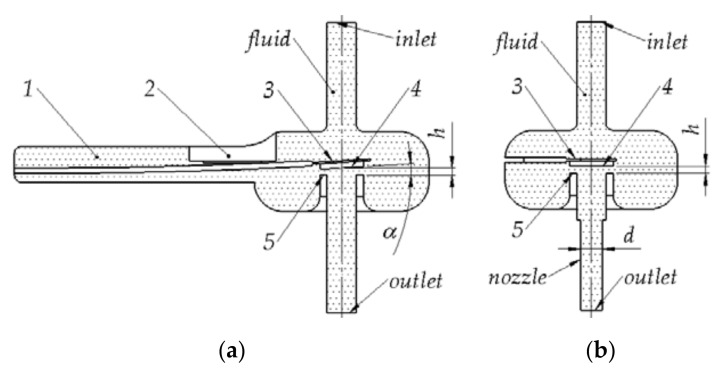
Fluid models used during CFD simulations: (**a**) base variant; (**b**) simplified variant; 1—buffer part; 2—limiter mount; 3—control plate; 4—control plate seal; 5—injector seat; *h*—opening range; *d*— nozzle outlet diameter; *α*—control plate rotate angle.

To perform CFD simulation, it was necessary to create a solid model of the fluid (Figure 2). Analysis was based on numerous electromechanical characteristics presented later in this paper, and the values of the *α* angle at the maximum openings of the injector valve were found to be in the range of up to 3° (Figure 2a). Therefore, it was decided to simplify the model (Figure 2b) by removing the buffer part (1) and the limiter mounting location (2), leaving only the control plate section (3) covered by the remaining fluid. The control plate was also simplified to a position parallel to the injector seat (5), which, in the authors’ opinion, will have little effect on the CFD simulation results. The valve opening in the simplified variant (Figure 2b) corresponded to the value measured from the injector seat (5) to the center of the control plate seal (4) of the initial variant (Figure 2a). Additionally, the installation of a nozzle outlet with diameter “*d*” was taken into account. The CFD analysis was aimed at determining the flow characteristics of the injector, which made it possible to determine the effective opening range of the valve.

The CFD software Ansys Fluent (Symkom—ANSYS Channel Partner, Warsaw, Poland) was used in the flow analysis. The computational algorithm was based on the Reynolds-averaged Navier–Stokes (RANS) equations [45]:

– For the principle of mass conservation (the continuity equation):

(1) $$\frac{\partial \rho }{\partial t}+\mathrm{div}\left(\rho u\right)=0$$

– For the principle of momentum and angular momentum conservation:

(2) $$\rho \frac{\partial \rho }{\partial t}=\rho F+\mathrm{div}S$$

– For the principle of energy conservation:

(3) $$\rho \frac{d}{dt}\left(TC_{v}+\frac{u^{2}}{2}\right)=\rho Fu+\rho q+\mathrm{div}\left(\Gamma \mathrm{grad}T\right)+\mathrm{div}\left(Su\right)$$

where *t* is time, *u* is fluid velocity, *ρ* is fluid density, *F* is body force for mass unit, *S* is stress tensor, *C_v_* is specific heat capacity with fixed volume, *T* is temperature, *q* is unit output of internal source of heat and Γ is thermal conductivity.

The equations of the turbulent kinetic energy (*k*) and specific dissipation rate (ω) for the SST turbulence model are obtained from a combination of *k*-*ε* and *k*-*ω* turbulence models [46]:

(4) $$\left\{\begin{array}{l} \frac{\partial \left(\rho k\right)}{\partial t}=\nabla \left[\left(\mu +\sigma_{k}\mu_{T}\right)\nabla k\right]+P_{k}-\beta^{*}\rho \omega k\\ \frac{\partial \left(\rho \omega \right)}{\partial t}=\nabla \left[\left(\mu +\sigma_{\omega }\mu_{T}\right)\nabla \omega \right]+\gamma \frac{\rho }{\mu_{T}}P_{k}-\beta \rho \omega^{2}+2\left(1-F_{1}\right)\rho \sigma_{\omega 2}\frac{\left(\nabla k\right)\left(\nabla \omega \right)}{\omega } \end{array}\right.$$

where *P_k_* is production term; *d_w_* is wall distance; *ρ* is density; *μ* is dynamic viscosity; *S* is strain rate; and *β*, *γ*, *σ* and *F* are functions and constants of the model.

**Figure 3:**
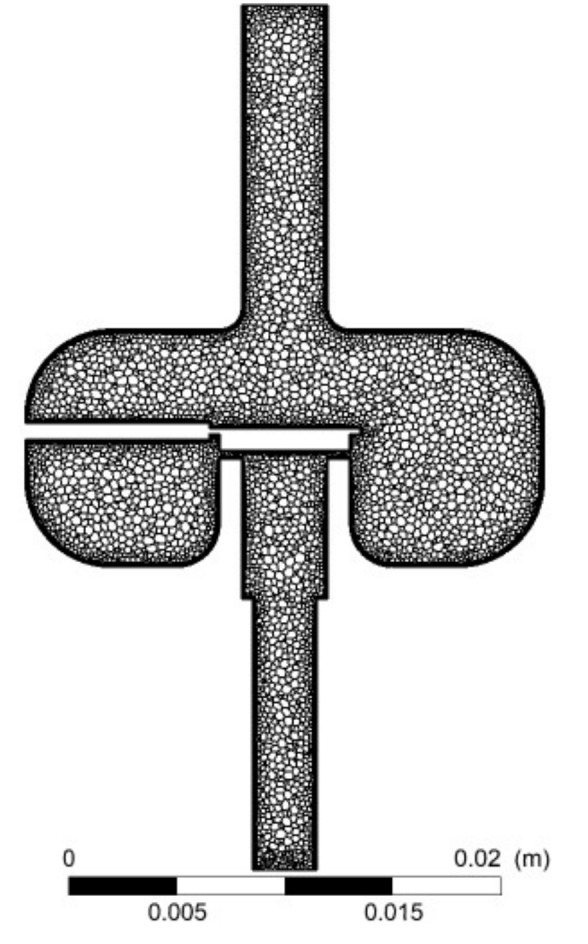
Example of polyhedral mesh in the longitudinal plane of the injector at the opening *h* = 0.4 mm and the outlet nozzle diameter *d* = 3 mm.

A tetrahedral mesh was created for the assumed solid model of the fluid according to Figure 2b. While creating the mesh, the following parameters related to its size were used: curvature normal angle 10°, min size 8 × 10^−4^ mm, max face size 0.2 mm, max tet size 0.4 mm and growth rate 1.2. Additionally, the inflation option with the following parameters was used: max layers 4 and growth rate 1.2, and the no-slip variant was added. Assessing the mesh qualitatively, it was found that each time with the number of elements of about 2 million, skewness was about 0.21 and orthogonal quality was 0.88. The obtained values of the mesh quality parameters can be considered satisfactory [47]. The tetrahedral mesh was then converted into a polyhedral mesh in Fluent solving, and the process was complemented with systematic cell optimization (Figure 3). Air was taken as the working medium. In the experimental studies, a low-pressure gas-phase injector was used for safety reasons. The use of air was considered appropriate in the context of further reference to the experimental results of other gas injectors.

The initial conditions were set as an inlet–outlet pressure difference of 1 × 10^5^ Pa, taking into account a turbulent intensity of 5% and turbulent viscosity ratio of 10. The solution method was set as a standard SIMPLE scheme. The control values of all residuals were assumed to be 1 × 10^−4^. The results obtained are presented in the last section.

### 2.3. The Electromechanical Characteristics of a Piezoelectric Transducer

**Figure 4:**
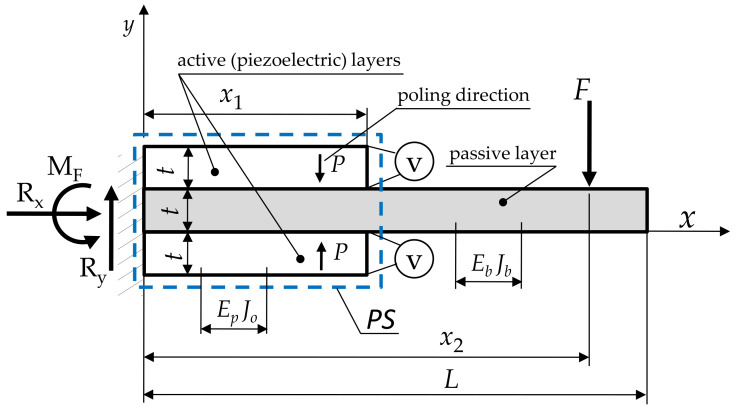
Cantilever converter of one PS.

To determine the electromechanical characteristics of the transducer (Figure 4), the method proposed in [48,49] was used. It consists of implementing the so-called piezoelectric segments (PSs) into the bent beam.

It allows the transducer to be treated as a homogeneous single-layer beam with a locally located three-layer PS that contains two components: piezoelectric (active) and nonpiezoelectric (passive). In the transducer (Figure 4), the left side is fixed, while the right side can move freely. The operating load comes from an external force *F* (generated by the gas-intake manifold pressure difference, occurring only in the closed state) and an electric moment *M_e_* caused by an applied voltage *V*. Based on the equilibrium conditions of forces and moments, the values of the reactions in the fixed support were determined; they are $R_{y}=F,R_{x}=0,\;M_{F}=Fx_{2}$.

A mathematical model for the bending of a beam with such a structure was developed under the following assumptions:

- The thicknesses of the beam and the piezoelectric segment are identical.
- The heights of the beam and the passive layer are the same.
- There is no intermediate layer at the interface between the components and there is no slippage.
- Bending of the transducer occurs according to Euler’s hypothesis with equal radii of curvature of the deformed components.
- There is a transverse piezoelectric effect 1-3 in the active layer, resulting in pure bending.
- Then, the constitutive equations were developed:

(5) $$\frac{\partial^{2}y}{\partial x^{2}}=M(x)/E_{b}J_{b}+Me\gamma \left(H\left[x\right]-H\left[x-x_{1}\right]\right)$$

where:

$H\left[x-x_{i}\right]$—Heaviside’s function [50];
$\gamma =\frac{E_{b}\;J_{b}\;(Me+M(x))\;-\;E_{p}\;J_{o}\;M\left(x\right)}{E_{b}\;J_{b}\;E_{p}\;J_{o}\;Me}$—a coefficient to take account of the change in stiffness at the location of the PS;
*E*_p_, *E*_b_—Young’s moduli of piezoelectric and passive elements;
$J_{b}=\frac{bt^{3}}{12}$—moment of inertia of the beam element;
$J_{o}=\frac{b\left(E_{b}t_{}^{3}+26E_{p}t_{}^{3}\right)}{12E_{p}}$—moment of inertia of PS segment [49];
$M(x)=-F\;x_{2}+F\;x-F\left(x-x_{2}\right)H\left[x-x_{2}\right]$—bending moment due to mechanical load;
$Me=-\frac{vd_{31}E_{p}t\left(E_{b}+26E_{p}\right)b}{E_{b}-22E_{p}}$—bending moment due to electric load [49];
*d*_31_—piezoelectric constant.

An important feature of the obtained solution, distinguishing it from the solution for a homogeneous beam with a constant cross-section, is considering the bending moment generated by the electric load. The solution obtained allows the electromechanical characteristics of the converter to be determined. After double integration of the differential Equation (5), the analytical form of the desired characteristic was obtained:

(6) $$y(x)=A_{1}v+B_{1}F$$

where:

$$A_{1}=-\frac{6d_{31}E_{p}(x^{2}H\left[x\right]-{\left(x-x_{1}\right)}^{2}H\left[x-x_{1}\right]}{t^{2}(E_{b}+22E_{p})}$$

$$\begin{array}{l} B_{1}=-\frac{2}{b\beta E_{b}t^{3}}\left\{\begin{array}{l} {\left(x-x_{2}\right)}^{3}\left(26E_{p}\left(H\left[x-x_{1},x-x_{2}\right]-H\left[x,x-x_{2}\right]\right)+\beta H\left[x-x_{2}\right]\right)+\\ 26E_{p}x^{2}H[x](x-3x_{2})-26E_{p}{\left(x-x_{1}\right)}^{2}H[x-x_{1}](x+2x_{1}-3x_{2})\\ -\beta x^{2}(x-3x_{2}) \end{array}\right\},\\ \beta =E_{b}+26\;E_{p}. \end{array}$$

The integration constants were determined on the basis of the following boundary conditions: $\frac{\partial y}{\partial x}\left(0\right)=0,y(0)=0$.

### 2.4. Geometric and Material Features of a Piezoelectric Transducer

In all analyses performed, the geometric dimensions of the piezoelectric transducer were assumed to be constant and are as follows:

- Passive layer length *L* = 46 mm;
- Passive/active layer width *b* = 15 mm;
- Active layer length *x*_1_ = 41 mm;
- Layer thickness *t* = 0.25 mm;
- Coordinate of the point at which the force *F* is applied *x*_2_ = 44.5 mm.

In the electromechanical tests, the individual components of the transducer were assumed to be made of typical materials used in MEMSs. The following materials were selected for passive elements: copper, nickel, silicon alloys and aluminum alloys. Active layers were assumed to be made of polymeric or ceramic piezoelectric materials. The materials used and the material data necessary to perform the analyses are presented in Table 1 and Table 2.

More detailed specifications of these materials can be found in [51] (passive materials) and [52] (piezoelectric materials). The results obtained are given below.

## 3. Results and Discussion

### 3.1. The Results of the Flow Test

**Figure 5:**
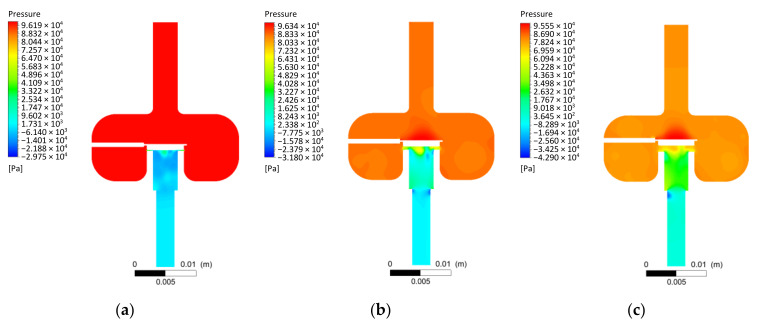
Example pressure field distributions for given CFD simulation conditions: (**a**) *h* = 0.1 mm; (**b**) *h* = 0.5 mm; (**c**) *h* = 1 mm.

Flow test results in the initial section are presented as a distribution of pressure fields and velocity waveform lines in the longitudinal plane of the injector showing the area of the control plate. The pressures are presented relative to the ambient pressure. The pressure field distributions (Figure 5) show the influence of the injector valve opening degree. At an opening of *h* = 0.1 mm, the pressure is significantly higher in the chamber above the control plate than in the chamber below the control plate, and local vacuums are visible just after passing through the valve. As the opening increases to *h* = 0.5 mm, this difference changes, and fields of local vacuums appear at the beginning of the outlet nozzle. At opening *h* = 1 mm, the pressure after the valve increases compared to the previous ones. After leaving the valve, the pressure increases, and local vacuums appear with a slight shift compared to the front of the outlet nozzle.

**Figure 6:**
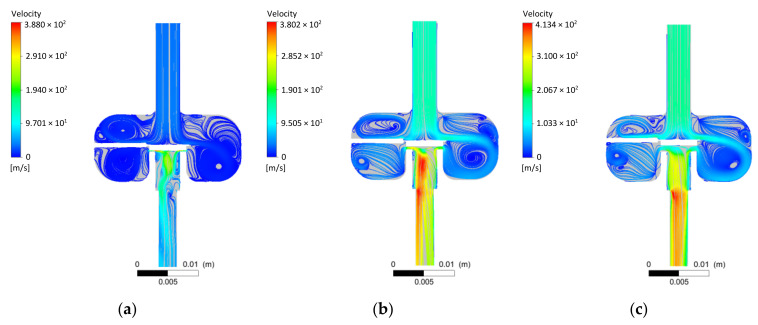
Example line distributions of velocity waveforms for given CFD simulation conditions: (**a**) *h* = 0.1 mm; (**b**) 0.5 mm; (**c**) 1 mm.

The streamlines shown in Figure 6 correlate with the pressure field descriptions. In the variant *h* = 0.1 mm, the highest flow velocities are located under the control plate seal, near the injector seat. For the opening *h* = 0.5 mm, the maximum flow velocities are reached in the part behind the valve and at the beginning of the outlet nozzle. Opening *h* = 1 mm causes maximum velocities to appear behind the front of the inlet nozzle.

**Figure 7:**
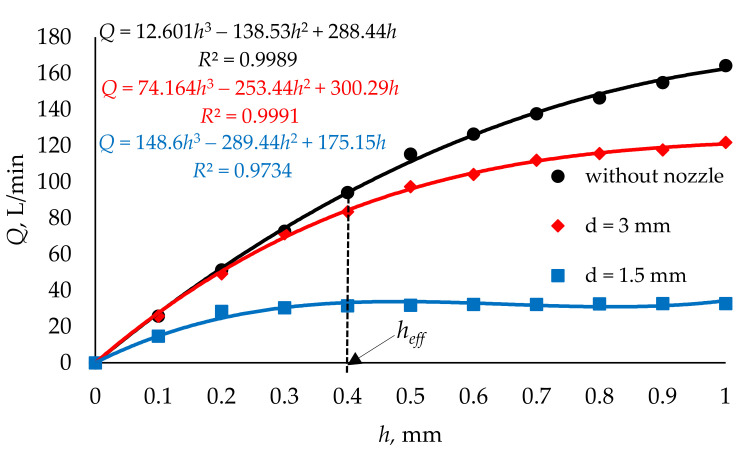
The flow characteristics of the injector with different outlet nozzle variants: without nozzle and with nozzles of different diameters.

Based on the volumetric flow rate read from the injector’s outlet surface, its flow characteristics were determined for different variants of the outlet port. These were variants without and with *d* = 1.5 and 3 mm nozzles. It can be seen (Figure 7) that there is a significant effect on the volumetric flow rate value caused by the nozzle diameter, which restricts the flow. For the nozzle diameter *d* = 1.5 mm, the volumetric flow rate stabilizes above the opening degree (valve lift) of the injector of *h* = 0.2 mm. In the case of nozzle diameter *d* = 3 mm, the flow rate changes only slightly above *h* = 0.6 mm of valve lift, while in the case of the variant without nozzle, the flow rate increases over the entire range tested. The maximum values of volumetric flow rate in the tested range (0–1 mm) were as follows: *d* = 1.5 mm—*Q* = 32.82 L/min; *d* = 3 mm—*Q* = 121.93 L/min; without nozzle (*d* = 4 mm)—*Q* = 164.31 L/min. The variant without nozzle is only a comparative one, as the low-pressure gas-phase injectors are assembled with nozzles, where the opening with diameter *d* = 3 mm is the maximum used in such systems, and the volumetric flow rate at the level of 80–100 L/min is the one required to supply the engine with gas [40,53]. The analysis of the characteristics presented in Figure 7 allowed the effective range of the injector opening to be defined as *h*_eff_ = 0.4 mm, which is similar to the piston injectors available on the market [54].

### 3.2. Electromechanical Studies Results

The aim of the electromechanical tests performed was to determine the energy consumption of the transducer when using various combinations of materials used for its components. By treating the piezoelectric layers as a capacitor, the total energy supplied to the transducer can be written as follows:

(7) $$E_{cap}=\frac{v^{2}x_{2}\;\varepsilon_{o}\varepsilon_{r}b}{t},$$

where *ε_o_* and *ε_r_* are the permittivity of the vacuum (8.854187817 × 10^−12^ C^2^/(N·m^2^)) and relative permittivity, respectively.

Using Formulas (6) and (7), the energy required to power the converter and the accompanying electrical voltage were determined for all variants of material combinations (40 different combinations in total). As already mentioned, the force *F* is caused by the pressure difference between the lower surface of the transducer and the valve seat. This force disappears as soon as the valve is opened. Thus, with an effective opening equal to *h_eff_* (Figure 7), the force *F* does not occur. The results obtained, determined at the effective opening of the valve (*y*(*x*_2_) = *h_eff_* = 0.4 mm) and at *F* = 0, are presented in Table 3.

To check the energy consumption of the transducer when it is opened (when the valve is unsealed), the necessary calculations were performed assuming that *y*(*x*_2_) *=* 0.01 mm and *F* = 1.245 N [40]. The obtained results are shown in Table 4.

From the results obtained, it can be concluded that all material combinations, except those containing PVDF polymer, can be used in the actuator. Moreover, it can be observed that the energy requirement (*E_cap_*), as well as the applied voltage V, varies with the material constants of the individual transducer components (Young’s moduli, relative permittivity, piezoelectric constant). It can be noted that the required current voltage, necessary to obtain the assumed deflection of the transducer *y*(*x*_2_), always increases with the decrease in the absolute value of the piezoelectric constant *d*_31_. A similar trend occurs for the energy demand, but its value is additionally influenced by the relative permittivity *ε_r_*—with the increase in this parameter, the *E_cap_* increases. As far as the influence of Young’s moduli is concerned, the use of materials with higher values of *E*_b_ or *E*_p_ generally results in the increase in current voltage and, consequently, energy consumption. The exception is the piezoelectric polymer PVDF, which is caused by the fact that this material is characterized by simultaneously small Young’s modulus and piezoelectric constant *d*_31_, so it is used for sensors rather than actuators (high current voltage is required to induce the deformation).

**Figure 8:**
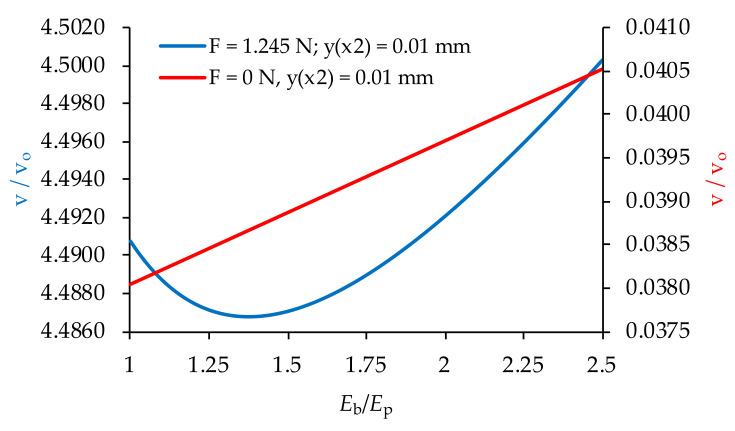
Variation of the voltage with the relative stiffness *E*_b_/*E*_p_, Vo = 100 V.

When analyzing the influence of Young’s moduli on the tested transducer performance, one should also consider the transducer relative stiffness—*E*_b_/*E*_p_. In the case of zero mechanical load (*F* = 0), achieving the desired deflection *y*(*x*_2_) requires the use of higher values of current voltage (Figure 8). The required voltage v increases proportionally to *E*_b_/*E*_p_. In the opposite case (*F* ≠ 0), an increase in the relative stiffness may result in an increase or decrease in the operating voltage v (Figure 8).

**Figure 9:**
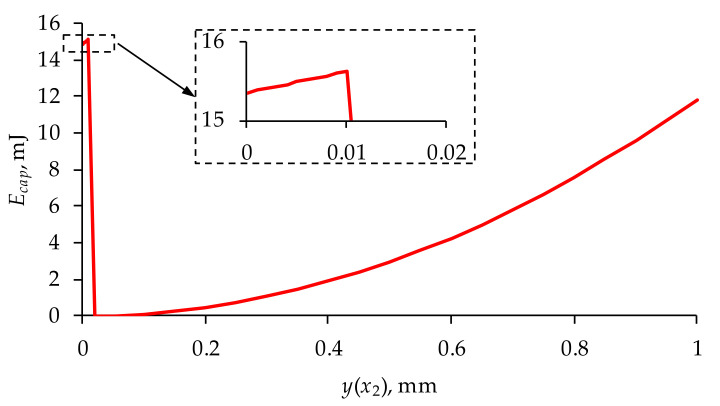
Energy demand diagram for the PTZ5H/silicon converter (*y*(*x*_2_) *=* 0.01 mm, *F* = 1.245 N—valve unsealing; *y*(*x*_2_) *= h_eff_ =* 0.4 mm, *F* = 0—achieving effective valve opening).

Analyzing the obtained result (Table 3 and Table 4), it was also found that both the energy consumption of *E_cap_* and the applied voltage *V* are the highest during valve unsealing, which is shown graphically in Figure 9.

**Figure 10:**
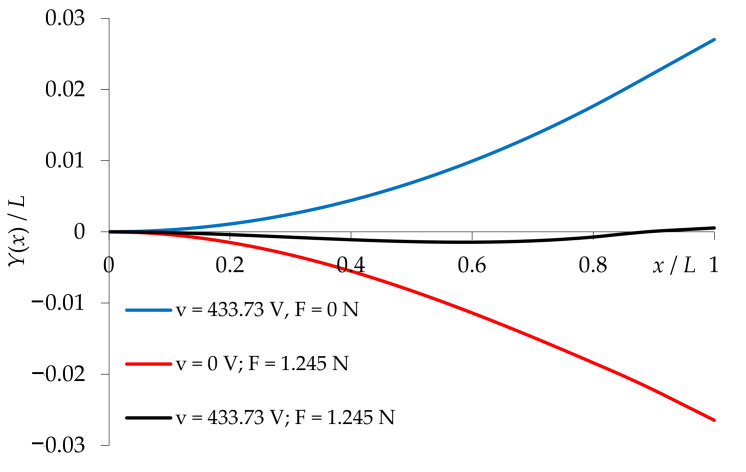
Electromechanical characteristics of the PTZ5H/silicon oxide converter (6).

Furthermore, it was found that the least energy-consuming variant is a transducer with an active component made of PTZH5 and a passive component made of silicon oxide. The electromechanical characteristics of this transducer are shown in Figure 10.

Analyzing the above characteristics, it can be concluded that in the case of applying an electrical and mechanical load simultaneously, the deflection of the transducer along its length may change the polarization. Thus, the stiffness and preload in the variant with a compression spring in the limiter (Figure 1) will depend on the materials used for the piezoelectric actuator.

The developed mathematical model describes an idealized converter having a perfect connection between the individual layers. In practice, converter components are joined together by adhesive bonding or soldering (after metal coating of the piezoelectric element in a vacuum) or are assembled under mechanical preload [55]. Thus, in physically existing converters, there is always some kind of intermediate layer in the planes of component connection. As a result, the deflection of the real beam transducer will differ from those determined by its analytical/numerical model. The discrepancy of the results may, in extreme cases, amount to several percent [56]. Therefore, the results presented above (energy demand, electric voltage) may be affected by a similar error.

## 4. Conclusions

The research conducted had two objectives. The first was to evaluate the SMs, which are piezoelectric materials, from the viewpoint of their applicability in the gas injector executive system. The actuator, which controls the opening and closing of the injector valve, was a piezoelectric cantilever, consisting of three layers—two active (piezoelectric ceramics/polymers) and one passive. For the passive layer, typical materials used in MEMSs (copper, nickel and silicon and aluminum alloys) were used. Another objective was to determine the energy consumption of a piezoelectric actuator with different material combinations of its components. Achieving the set goals required both flow and electromechanical analyses. These analyses were carried out for an injector, the conceptual solution of which was previously patented by the authors.

Based on the CFD flow studies results, it was concluded that:

- The initial tetrahedral mesh used each time had about 2 million elements; the skewness was about 0.21 and the orthogonal quality was close to 0.88. The Ansys Fluent software used for the calculations converted the tetrahedral mesh into a polyhedral mesh. In a further step using the in-solver SIMPLE scheme, significant flow parameters were determined with control values of all residuals being 1 × 10^−4^.
- The distributions of the pressure fields in the longitudinal section of the low-pressure gas-phase injector obtained from the flow tests showed a significant influence of the opening stage. The pressure difference in the areas upstream and downstream of the injector valve and the local vacuum zones were thus identified. The streamlines presented for the three cases of injector valve opening stages correlated with the pressure fields.
- CFD tests enabled the determination of flow characteristics of the low-pressure gas-phase injector. Three variants of the injector outlet diameter significantly differentiated the characteristics in question. Maximum values of volumetric flow rate in the examined range (0–1 mm) were as follows: *d* = 1.5 mm—*Q* = 32.82 L/min; *d* = 3 mm—*Q* = 121.93 L/min; without nozzle (*d* = 4 mm)—*Q* = 164.31 L/min
- Analysis of the flow characteristics made it possible to determine the effective degree of valve opening at *h*_eff_ = 0.4 mm. This was based on the volumetric flow rate variation.

On the basis of the electromechanical tests carried out on the transducer, which constitutes the injector executive system, it was found that:

- All material combinations, except those containing PVDF polymer, can be used in the actuator.
- The energy consumption is influenced by the material parameters of all transducer components. Generalizing, it can be said that it increases as the absolute value of the piezoelectric constant *d*_31_ decreases and the relative stiffness increases.
- The least energy-consuming design was the variant in which the active element of the transducer is made of PTZH5 and the passive element is made of silicon oxide.
- The energy consumption during valve unsealing is greater than that required to achieve effective valve opening.

The performed flow and electromechanical tests allow the conclusion of the functional correctness of the low-pressure gas-phase injector and at a later stage may be the basis for making a prototype to confirm theoretical analyses. In further studies, the authors plan to present a dynamic model of the piezoelectric actuator operation and a combined flow model with a movable flow control element.

## Figures and Tables

**Table 1:** Materials used for the passive components of the transducer [51].

Passive Materials								
	**Aluminum**	**Silicon Oxide**	**Silicon**	**Copper**	**Nickel**	**Silicon Nitride**	**Aluminum Oxide**	**Silicon Carbide**
*E*_b_ (GPa)	69	73	129	135	207	304	393	430

**Table 2:** Materials used for the active components of the transducer [52].

	Piezoelectric Materials				
	Polymer	Ceramic			
		Soft		Hard	
	PVDF	PTZ5H	APC856	APC841	PTZ8
*E*_p_ (GPa)	3	62.1	66.6	85.4	86.9
*d*_31_ (pC/N)	23	−320	−260	−109	−97
*ε_r_*	12	3400	4100	1350	1100

**Table 3:** Energy demand *E_cap_* and electric voltage v determined for effective valve opening (*y*(*x*_2_) *= h =* 0.4 mm, *F* = 0).

		Piezoelectric Materials				
		PVDF	PTZ5H	APC856	APC841	PTZ8
**Passive MEMS materials**	**Aluminum**	4.87 *	1.88 *	3.41 *	6.26 *	6.44 *
		4142.36 **	152.91 **	187.65 **	443.02 **	497.52 **
	**Silicon Oxide**	5.16 *	1.89 *	3.43 *	6.29 *	6.46 *
		4265.10 **	153.34 **	188.07 **	443.93 **	498.53 **
	**Silicon**	10.16 *	2.04 *	3.68 *	6.66 *	6.83
		5983.42 **	159.30 **	194.92 **	456.67 **	512.59
	**Copper**	10.79 *	2.06 *	3.71	6.70 *	6.87 *
		6167.52 **	159.94 **	195.65 **	458.03 **	514.10 **
	**Nickel**	19.91 *	2.26	4.05 *	7.18 *	7.37 **
		8376.78 **	167.61 **	204.46 **	474.41 **	532.18 **
	**Silicon Nitride**	36.57 *	2.55	4.54 *	7.87 *	8.05 *
		11,353.15 **	177.95 **	216.32 **	496.47 **	556.55 **
	**Aluminum Oxide**	56.27 *	2.83 *	5.00 *	8.52 *	8.74 *
		14,084.04 **	187.75 **	227.20 **	516.71 **	579.66 **
	**Silicon Carbide**	68.39	2.94 *	5.20 *	8.80 *	9.00 *
		15,526.20 **	191.37 **	231.72 **	525.13 **	588.20 **

*—energy stored *E_cap_* (mJ), **—voltage *V* (V).

**Table 4:** Energy demand *E_cap_* and electric voltage v determined at the moment of valve unsealing (*y*(*x*_2_) *=* 0.01 mm, *F* = 1.245 N).

		Piezoelectric Materials				
		PVDF	PTZ5H	APC856	APC841	PTZ8
**Passive MEMS materials**	**Aluminum**	4949.24 *	15.13 *	24.07 *	27.67 *	27.51 *
		132,083.49 **	433.80 **	498.29 **	931.07 **	1028.59 **
	**Silicon Oxide**	4972.49 *	15.12	24.06 *	27.64 *	27.48 *
		132,393.35 **	433.73	498.18 **	930.60 **	1028.05
	**Silicon**	5208.23 *	15.16 *	24.08 *	27.54 *	27.37 *
		135,495.35 **	434.26 **	498.47 **	928.91 **	1026.00 **
	**Copper**	5226.50 *	15.17 *	24.10 *	27.54 *	27.38 *
		135,732.80 **	434.41 **	498.61 **	929.00 **	1026.09 **
	**Nickel**	5388.43 *	15.32 *	24.31 *	27.69 *	27.52 *
		137,819.44 **	436.54 **	500.81 *	931.48 **	1028.70 **
	**Silicon Nitride**	5514.44 *	15.54 *	24.64 *	27.98 *	27.80 *
		139,421.67 **	439.73 **	504.22 **	936.29 **	1033.89 **
	**Aluminum Oxide**	5587.22 *	15.75 *	24.95 *	28.26 *	28.08 *
		140,338.68 **	442.67 **	507.33 **	940.97 **	1039.16 **
	**Silicon Carbide**	5616.46 *	15.83 *	25.07	28.38 *	28.19 *
		140,705.35 **	443.72 **	508.58 **	942.91	1041.10 **

*—energy stored *E_cap_* (mJ), **—voltage *V* (V).

## Data Availability

The data presented in this study are available on request from the corresponding author. At the time the project was carried out, there was no obligation to make the data publicly available.
